# Evaluating skin tone scales for dermatologic dataset labeling: a prospective-comparative study

**DOI:** 10.1038/s41746-025-02245-2

**Published:** 2025-12-22

**Authors:** Vanessa R. Weir, Yingjoy Li, Maura C. Gillis, Nicholas R. Kurtansky, Trina Salvador, Allan C. Halpern, Kelly C. Nelson, Jenna C. Lester, Veronica Rotemberg

**Affiliations:** 1 Dermatology Service, Division of Subspecialty Medicine, Department of Medicine, Memorial Sloan Kettering Cancer Center, New York, NY USA; 2 Beth Israel Deaconess Medical Center, Boston, MA USA; 3 Department of Dermatology, Division of Internal Medicine, University of Texas MD Anderson Cancer Center, Houston, TX USA; 4 Department of Dermatology, University of California, San Francisco, San Francisco, CA USA

**Keywords:** Cancer, Computational biology and bioinformatics, Diseases, Health care, Mathematics and computing, Medical research

## Abstract

Skin tone affects artificial intelligence (AI) performance in dermatology. While labeling datasets for skin tone could improve algorithm generalizability for detecting dermatologic malignancies, large-scale validation of skin tone assessments is lacking. This prospective observational study assessed reliability of subjective tools (Fitzpatrick Skin Type [FST], Monk Skin Tone [MST], Pantone SkinTone Guide) and an objective colorimeter for in-person and photography-based settings to evaluate utility for labeling dermoscopic datasets. Colorimetry (gold standard for color measurement) demonstrated high precision with in-person measurements. Of subjective scales, MST demonstrated slightly tighter clustering in the color space and high repeatability for in-person and photography-based assessments (latter varied by lighting). Dermoscopic image-extracted color values correlated poorly with colorimetry values. For subjective ratings, MST more effectively captured differences in AI melanoma classification scores than FST. Findings underscore that FST is not a proxy for skin tone; an important role remains for skin tone assessment to improve AI performance.

## Introduction

Non-invasive artificial intelligence (AI) tools have demonstrated significant potential in diagnosing skin cancers, offering a potential adjunct or alternative to traditional biopsy-based approaches^1,2^. However, skin tone is known to affect the accuracy of AI models used in clinical dermatology^3,4^. State-of-the-art, image-based AI algorithms have been shown to underperform when classifying skin lesions in individuals with darker skin tones, which may exacerbate existing disparities by increasing the risk of misdiagnosis and poor outcomes when applied in real-world clinical settings^4–6^. A key challenge in improving algorithmic performance stems from the lack of skin tone labels within the large datasets used to train these algorithms and benchmark their performance^3^. Incorporating skin tone labels into image datasets is a critical step toward improving the performance of AI tools in dermatologic care.

Measuring skin tone is complex, with a wide variety of methods available for its estimation and quantification. There are both subjective and objective measures for quantification. The Fitzpatrick Skin Type (FST) scale is currently the most widely used scale in dermatology and machine learning, although it was designed to categorize photosensitivity and does not measure skin tone^7,8^. Other visual tools, such as the Monk Skin Tone (MST) scale with 10 shades and the Pantone SkinTone Guide (Pantone [Pantone LLC, Carlstadt, NJ]) with 138 shades of varying undertones and pigmentation, estimate a range of skin tones through direct comparison with standardized color swatches^9–13^. While FST, MST, and Pantone are subjective and rely on human interpretation, colorimetric devices are designed to enable precise and objective quantification of skin color directly from the skin’s surface^14,15^. This study aimed to assess the reliability of measuring skin tone using these tools in both in-person and photography-based settings. We also compared subjective assessment tools (FST, MST, and Pantone) with objective colorimeter measurements and compared AI classifier scoring of benign lesions by the FST and MST scales.

## Results

### Patient cohort and skin site assessment

This prospective, single-center observational study included 64 participants recruited from dermatology clinics at a tertiary cancer care center who underwent full-body skin examination and imaging (dermoscopy and 3-dimensional [3D] total body photography [TBP]). For each participant, 5–13 skin lesions and 11 standardized non-lesional body sites were assessed for skin tone in person with MST, Pantone, and a colorimeter device. FST was determined by a board-certified dermatologist based on a photosensitivity questionnaire administered in clinic^7^. The patient cohort was intentionally recruited to ensure relatively equal distribution across all FST categories (I-VI) (Table 1).

**Table 1:** Patient-level (*n* = 64) and site-level (*n* = 1329) characteristics by Fitzpatrick Skin Type

	Fitzpatrick Skin Type (FST)						
*Patient-level data*							
	**I (15.6%,** ***n*** = **10)**	**II (18.8%,** ***n*** = **12)**	**III (17.2%,** ***n*** = **11)**	**IV (15.6%,** ***n*** = **10)**	**V (17.2%,** ***n*** = **11)**	**VI (15.6%,** ***n*** = **10)**	**Overall (*n*** = **64)**
Race							
White	10 (100.0%)	12 (100.0%)	11 (100.0%)	4 (40.0%)	1 (9.1%)	0 (0%)	38 (59.4%)
Black	0 (0%)	0 (0%)	0 (0%)	2 (20.0%)	8 (72.7%)	9 (90.0%)	19 (29.7%)
Asian	0 (0%)	0 (0%)	0 (0%)	1 (10.0%)	2 (18.2%)	1 (10.0%)	4 (6.3%)
American Indian	0 (0%)	0 (0%)	0 (0%)	1 (10.0%)	0 (0%)	0 (0%)	1 (1.6%)
Other	0 (0%)	0 (0%)	0 (0%)	2 (20.0%)	0 (0%)	0 (0%)	2 (3.1%)
Ethnicity							
Hispanic	0 (0%)	0 (0%)	0 (0%)	5 (50.0%)	1 (9.1%)	0 (0%)	6 (9.4%)
Dermoscopy device							
iPod Touch (7th Generation)	5 (50.0%)	7 (58.3%)	5 (45.5%)	5 (50.0%)	4 (36.4%)	5 (50.0%)	31 (48.4%)
Canon EOS Rebel (Single Lens Reflex)	5 (50.0%)	5 (41.7%)	6 (54.5%)	5 (50.0%)	7 (63.6%)	5 (50.0%)	33 (51.6%)
Measured with colorimeter	6 (60.0%)	6 (50.0%)	8 (72.7%)	8 (80.0%)	9 (81.8%)	10 (100.0%)	47 (73.4%)
*Site-level data*							
	**I (*n*** = **211)**	**II (*n*** = **250)**	**III (*n*** = **229)**	**IV (*n*** = **208)**	**V (*n*** = **223)**	**VI (*n*** = **208)**	**Overall (*n*** = **1329)**
Whole cohort							
Lesional	102 (15.8%)	121(18.8%)	112 (17.4%)	102 (15.8%)	103 (16.0%)	104 (16.1%)	644 (48.5%)
Non-Lesional	109 (15.9%)	129 (18.8%)	117 (17.1%)	106 (15.5%)	120 (17.5%)	104 (15.2%)	685 (51.5%)
Sites included in in-person reliability analysis							
MST and Pantone: Lesional	94 (15.5%)	111 (18.3%)	102 (16.8%)	95 (15.7%)	102 (16.8%)	103 (17.0%)	607 (45.7%)
MST and Pantone: Non-Lesional	99 (15.5%)	118 (18.5%)	105 (16.4%)	96 (15.0%)	120 (18.8%)	101 (15.8%)	639 (48.1%)
Colorimeter: Non-Lesional	66 (13.1%)	66 (13.1%)	85 (16.9%)	84 (16.7%)	98 (19.5%)	103 (20.5%)	502 (37.8%)
Sites included in objective colorimeter vs. subjective scales analysis							
Non-Lesional	66 (13.2%)	66 (13.2%)	83 (16.6%)	85 (17.0%)	98 (19.6%)	103 (20.6%)	501 (37.7%)
Sites included in photography-based reliability analysis							
MST - Cross-Polarized: Non-Lesional	99 (17.6%)	107 (19.0%)	116 (20.6%)	77 (13.7%)	88 (15.6%)	76 (13.5%)	563 (42.4%)
MST - White Light: Non-Lesional	99 (17.5%)	118 (20.8%)	108 (19.1%)	77 (13.6%)	77 (13.6%)	87 (15.4%)	566 (42.6%)
ITA - Image-Extraction: Non-Lesional	65 (13.7%)	65 (13.7%)	73 (15.4%)	78 (16.5%)	95 (20.0%)	98 (20.7%)	474 (35.7%)
Crowdsourced FST: Lesional	101 (16.3%)	118 (19.1%)	101 (16.3%)	100 (16.2%)	97 (15.7%)	101 (16.3%)	618 (46.5%)
Sites included in ADAE analysis							
MST and FST: Lesional	92 (15.7%)	110 (18.7%)	101 (17.2%)	91 (15.5%)	94 (16.0%)	99 (16.9%)	587 (44.2%)

*MST* Monk Skin Tone, *Pantone* Pantone SkinTone Guide, *ITA* individual typology angle, *ADAE* All Data Are Ext algorithm.

In total, 1329 skin-sites (644 lesional and 685 non-lesional) were included in the study for imaging and skin tone assessment. Cohorts for each of the following analyses are described in Table 1.

### Reliability of in-person skin tone assessment tools

**Fig. 1:**
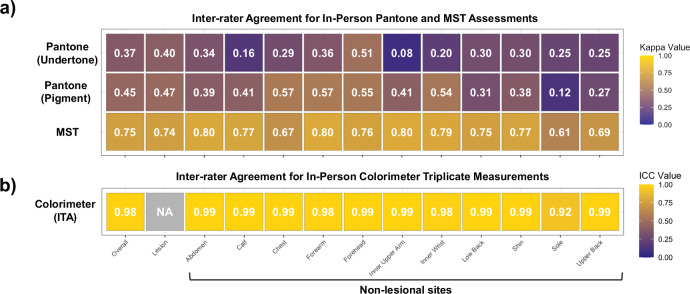
Inter-rater agreement for in-person skin tone assessment tools. Heatmap demonstrating inter-rater agreement (linear weighted Cohen’s kappa) for in-person assessment of skin tone using **a** Pantone SkinTone Guide (Pantone) undertone (5R to 5Y), Pantone pigment (1–15), and the Monk Skin Tone (MST) scale (1–10) across various body sites. **b** Heatmap demonstrating agreement (interclass correlation coefficient [ICC]) between triplicate colorimeter measurements of individual typology angle (ITA). Agreement was calculated for lesions only, each non-lesional site, and overall (including lesional and non-lesional sites).

In-person skin tone assessments performed by 2 independent raters in the clinic demonstrated varying levels of agreement across different body sites for both Pantone and MST (Fig. 1a). In-person MST assessment demonstrated substantial all-site agreement (linear weighted Cohen’s kappa [κ]: 0.75). Agreement was near-perfect at the abdomen (κ: 0.80), inner upper arm (κ: 0.80), and forearm (κ: 0.80), with at least substantial agreement across all other body sites. By comparison, the Pantone scale exhibited significantly lower agreement for both undertone (κ: 0.37) and pigment (κ: 0.45) assessments across all sites. Triplicate colorimeter measurements of individual typology angle (ITA) achieved near perfect reliability across non-lesional sites overall (intraclass correlation coefficient [ICC]: 0.98) (Fig. 1b; See Methods for ITA computation).

### Objective colorimeter vs. subjective FST, MST, and Pantone

**Fig. 2:**
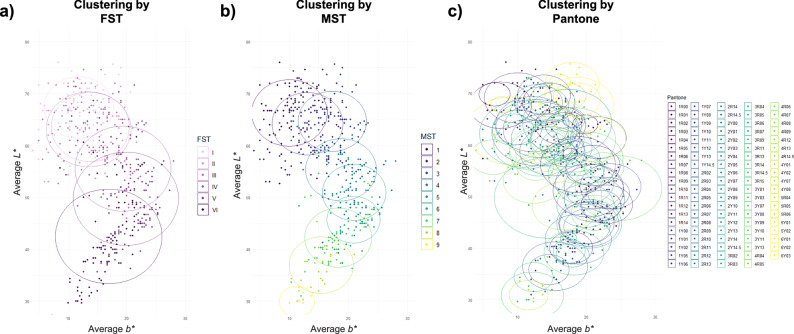
Clustering analysis to evaluate distinct groupings of in-person subjective Fitzpatrick Skin Type (FST), Monk Skin Tone (MST), and Pantone SkinTone Guide (Pantone) assessments within the CIELAB color space. The 2D CIELAB color space was created using the average L* (luminance) and b* (yellow chromaticity) of colorimetry measurements of non-lesional sites. A clustering analysis was performed for **a** in-person FST, **b** in-person MST (randomly selected from Rater 1 and Rater 2’s measurements), and **c**, in-person Pantone measurements (randomly selected from Rater 1 and Rater 2’s measurements) of the same sites. Each dot represents the colorimeter measurement (L* by b*) of a non-lesional site and is color-coded by in-person skin tone assessment categories. The overlapping circles represent the centroid for each assessment category. The radii of these circles correspond to the standard deviation of the Euclidean distance of objects within a cluster to their centroid (standard deviation of intra-cluster distance).

Overall, MST assessments demonstrated slightly better clustering than FST and Pantone, according to the CIELAB (L*a*b*) color space (Davies-Bouldin Index [DBI]: 2.70), particularly for sites rated MST 5 to 9 (Fig. 2a). In contrast, Pantone categories demonstrated more dispersion (DBI: 7.59), and FST clustered the worst (DBI: 10.59), especially for FST IV to VI (Fig. 2b, c; per-cluster dispersion measures are described in Supplementary Table S1). However, the degree of clustering according to Rousseeuw’s Silhouette Index (RSI) was close to 0 for all 3 scales, implying weak clustering for all subjective forms of skin tone measurement. Because lighting, vascularity, and surface-texture may differ across anatomical regions, Supplementary Fig. S1a, b presents the cluster dispersion measures separately for different body sites. According to DBI, FST-specific classes exhibited the weakest clusters in CIE (L*, b*) space for most analyzed body sites. According to RSI, Pantone classes exhibited the weakest clustering in all sites. When considering both metrics, MST classes seemed to be better associated with colorimeter-derived CIE (L*, b*) as compared to FST classes and Pantone classes.

### Photography-based skin tone labeling

#### In-person MST vs. 3D TBP-based MST

**Fig. 3:**
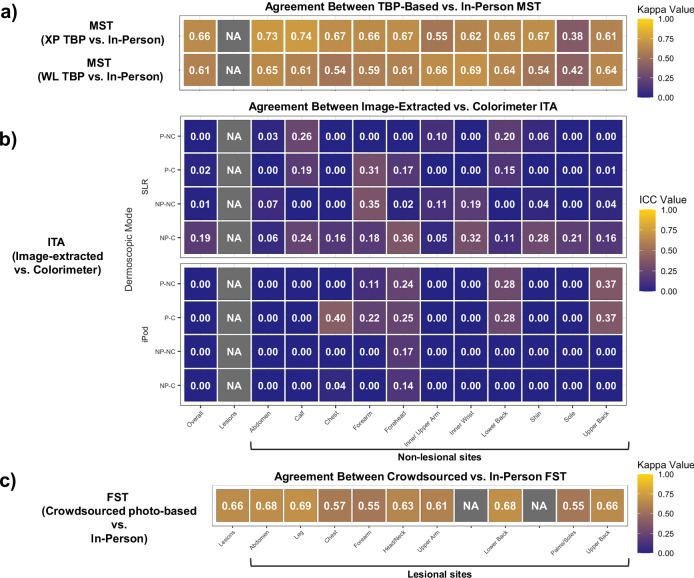
Agreement between photography-based skin tone assessments compared with in-person assessments. **a** Heatmap demonstrating agreement (linear weighted Cohen’s kappa [κ]) between total body photography (TBP)-based Monk Skin Tone (MST) assessment of non-lesional sites compared with in-person evaluation, under cross-polarized (XP) and white light (WL) settings. **b** Heatmap demonstrating agreement (interclass correlation coefficient [ICC]) between in-person colorimeter measurements of individual typology angle (ITA) values for non-lesional sites and median pixel-wise ITA values extracted from dermoscopy images captured with 2 dermoscopic devices (iPod and SLR) under 4 dermoscopy modes: polarized non-contact (P-NC), polarized contact (P-C), non-polarized non-contact (NP-NC), and non-polarized contact (NP-C). **c** Heatmap demonstrating agreement (linear weighted κ) between in-person Fitzpatrick Skin Type (FST) and crowdsourced dermoscopy-based FST labeling of lesional sites. Lesional sites were mapped as closely as possible to general anatomical categories corresponding to standardized non-lesional sites.

Overall, MST assessments of non-lesional body sites using cross-polarized (XP) and white light (WL) 3D TBP demonstrated substantial agreement with in-person assessment (κ: 0.66 and κ: 0.61, respectively) (Fig. 3a). The highest agreement was on the calf (κ: 0.74) and abdomen (κ: 0.73) under XP lighting, and on the inner wrist (κ: 0.69), inner upper arm (κ: 0.66), and abdomen (κ: 0.65) under WL. For both XP and WL, the lowest agreement with in-person skin tone assessment was on the sole (XP κ: 0.38, WL κ: 0.42). Compared with in-person MST labels, WL TBP-based ratings were significantly lower (mean difference: -0.70, 95% confidence interval [CI]: –0.79 to −0.61, *P* < 0.001), indicating systemic negative bias. In contrast, XP-based ratings were slightly but significantly higher than in-person ratings (mean difference: +0.15, 95% CI: +0.06 to +0.23, *P* = 0.0012) (Supplementary Fig. S2a, b).

#### Colorimeter vs. image-extracted ITA

We assessed whether median ITA values extracted from dermoscopic images aligned with in-person colorimeter-derived ITA. Overall, there was very poor agreement (ICC: 0.00–0.19) between the colorimeter-derived ITA values and those estimated from images across all 4 dermoscopy modes, including polarized non-contact (P-NC), polarized contact (P-C), non-polarized non-contact (NP-NC), and non-polarized contact (NP-C) (Fig. 3b). There were demonstrable shifts in luminance and yellow chromaticity between image-extracted L* and b* versus colorimeter-derived values depending on dermoscopy lighting (polarized and non-polarized modes) (Supplementary Fig. S3).

#### In-person FST vs. crowdsourced photography-based FST

To investigate whether FST could be estimated from dermoscopy images alone, we collected crowdsourced labels using DiagnosUS (Centaur Labs, Boston, MA, USA; https://www.diagnosus.com/), a mobile application that gamified this task for annotators. The overall agreement (κ) of crowdsourced FST labeling, as compared with in-person FST, was 0.66. Agreement was the highest at the leg (κ: 0.69), abdomen (κ: 0.68), and lower back (κ: 0.68) (Fig. 3c). The overall concordance between crowdsourced FST labeling and in-person FST determined by dermatologists was 55.8%. Concordance was the highest for FST VI (79.6%) and I (74.2%), but the lowest for IV (36.1%) and II (46.3%) (Supplementary Fig. S2c). Chi-squared analyses revealed that crowdsourced FST label accuracy also varied significantly by anatomical site and dermoscopy lighting mode (both *P* < 0.001), as visualized in the example image panels in Supplementary Fig. S4a, b.

### Fairness assessment for benign lesion classification

**Fig. 4:**
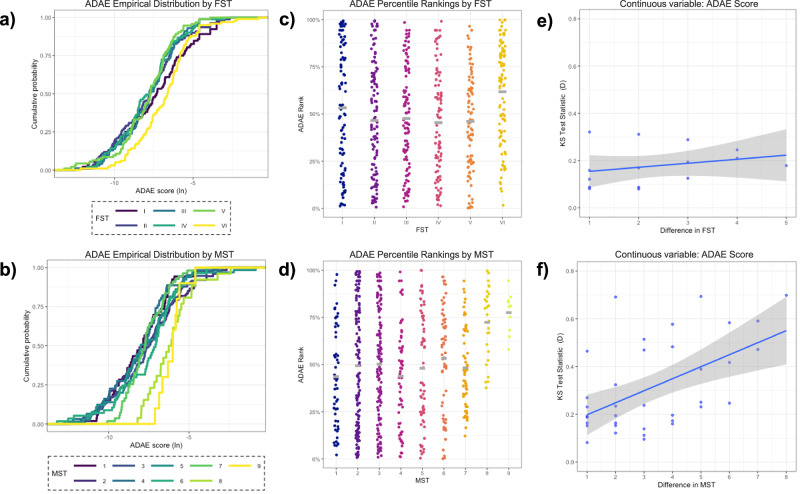
Classification of benign lesions using a melanoma classifier (All Data Are Ext algorithm; ADAE) stratified by Fitzpatrick Skin Type (FST) and Monk Skin Tone (MST). **a** Cumulative distribution curves of log-transformed ADAE scores stratified by FST. Differences in score distributions are observed across FST groups. **b** Cumulative distribution curves of ADAE scores stratified by MST shades. MST shades provide finer granularity, demonstrating more distinct score distributions compared with FST categories. **c** ADAE score percentile rankings for FST groups. Variability in mean scores is noted, with heterogeneity increasing across the FST spectrum. **d** ADAE score percentile rankings for MST shades. Higher mean scores are observed for MST shades 8-9, with MST providing a more nuanced classification of skin tone compared with FST. **e** Kolmogorov–Smirnov (KS) test statistics for ADAE scores by differences in FST groups. KS statistics increase slightly with greater differences between FST classes. **f** KS test statistics for ADAE scores by differences in MST shades. KS statistics increase substantially with greater differences in MST shades compared with FST, suggesting that MST captures score distribution differences more effectively.

P-C dermoscopy images of lesional sites were analyzed by a state-of-the-art open-source algorithm (ADAE: “All Data Are Ext”), which provides a malignancy risk score (0–100%) to each image where scores closer to 0% are more likely to be benign and those closer to 100% are more likely melanoma^16–19^. Algorithm score distributions were compared across FST and MST categories (Fig. 4).

Log-transformed cumulative distribution curves of benign lesion scores revealed that score distributions were more similar when stratified by FST (Fig. 4a) and more disparate when stratified by MST (Fig. 4b). Distribution of ADAE scores was similar across FST categories except for VI (Fig. 4c). Distribution of ADAE scores was also similar across MST groups except for MST 8 and 9 (Fig. 4d). The Kolmogorov–Smirnov (KS) statistic was calculated to investigate differences in algorithm score distributions between skin tone classes (Supplementary Table S2a, b). These differences increased with greater differences between FST (Fig. 4e) and MST (Fig. 4f) classes, indicating larger dissimilarities in algorithm malignancy risk score distributions across more disparate skin tone groups. Notably, the MST scale showed a more pronounced increase in the KS statistic as the difference in shade increased compared with the FST classifications, suggesting that MST may be better than FST at capturing differences in algorithm score distribution among subjective scales.

## Discussion

This study provides the first direct comparison of subjective (FST, MST, and Pantone) and objective (colorimeter) skin tone assessment tools for labeling dermatologic datasets, highlighting both the relative strengths and persistent limitations of each method. While our findings suggest that colorimetry may demonstrate promising reliability for skin tone assessment, no single approach proved consistently generalizable across all contexts. These results highlight the need for continued refinement, transparency, and validation of skin tone assessment methods for labeling dermatologic datasets.

Reliable skin tone labeling is essential to ensure the accuracy of AI-based dermatologic tools^3^. Despite evidence of poorer performance in darker skin tones, there are no established guidelines on how best to measure skin tone for image datasets^4^. Defining skin tone is challenging; although typically framed as the perception of major chromophores, such as melanin and hemoglobin, many contextual factors influence skin tone perception and measurement including sociocultural context, lighting, hair, anatomical location, sun damage, and vascularity^10^. To inform effective use of AI in dermatology, labeling methods should exhibit (i) construct validity, (ii) generalizability, and (iii) a capacity to detect disparate AI performance. In this study, we prospectively curated one of the first dermatological image datasets spanning a wide range of skin tones, uniquely labeled using multiple skin tone assessment tools. By evaluating both in-person and photography-based labeling methods, our findings highlight the strengths and limitations of each approach and underscore the need for more reliable, retrospective-compatible labeling strategies and scales to enhance the utility of dermatologic AI algorithms across diverse skin tones.

In-person skin tone labeling tools must be able to precisely capture variations in skin color to enable robust evaluations of AI performance. Although FST has been adapted as the de facto “gold standard”, this scale was designed based on subjective questionnaires to classify the photosensitivity of different skin types^7,20,21^. Recent studies have demonstrated that FST is often inaccurately conflated with objective skin color, a limitation that is central to our work^7,22,23^. Our findings underscore this discrepancy: FST categories exhibited the weakest clustering in colorimetry-based analyses overall, suggesting that each FST category spans a wide range of true skin color compared with MST and Pantone. Similar findings regarding poor correlations between FST phototypes and objective colorimeter-derived values have been noted in prior work^8^. Because we used the patients’ reported tanning and burning propensity to determine FST, we were unable to directly compare its reliability against visually based skin tone scales such as MST or Pantone.

In contrast, MST’s palette of 10 discrete shades was conceived by sociologists for ease of labeling images but is still in the process of being prospectively validated in different contexts^9–11,24–26^. In our in-person assessments, MST achieved an overall inter-rater agreement of 0.75; however raters were localized to one cultural context, which is known to affect MST ratings^11^. Agreement was especially high at the abdomen, inner upper arm, and forearm. Inter-rater reliability, while high for in-person assessment, does not ensure accuracy, especially in the absence of a definitive ground truth, as skin tone perception remains subjective and context-dependent. For instance, certain anatomical sites, particularly those prone to shadowing or other skin features (i.e., solar lentigines, callused areas, increased vascularity), showed lower agreement. Moreover, MST still demonstrated weak clustering overall, highlighting areas of improvement for this scale. There is also a lack of publicly available documentation that has explicitly described how MST shades were selected or spaced. Its reported validation has focused on perceived inclusivity in a multi-ethnic cohort, with no correlation to objective “ground truth”^27^. Lastly, Pantone’s SkinTone Guide, while theoretically covering a broader spectrum than MST (including variations in erythema), showed diminished labeling consistency, likely due to the number of swatches.

Given that categorical scales may not capture the full range of human skin tone, more multidimensional approaches have been explored^23^. Colorimetry enables more objective, standardized measurement of skin color by quantifying continuous variables such as luminance (L*), chromaticity (a*, b*), ITA derived from L*a*b*, along with erythema and melanin indices. Unlike subjective methods reliant on color swatches, colorimetry minimizes human bias and lighting inconsistencies by creating a controlled measurement environment, yielding high test-retest reliability across anatomical sites in our cohort and in other studies^28,29^. However, it is important to note that the ITA classification system was first developed from a sample of over 35,000 skin tone measurements collected exclusively from individuals of European ancestry. Its original categorization into 4 skin tone bins (e.g., “very light” to “tan”), although later expanded to include bins for “dark” and “very dark” skin, was not designed for racially diverse populations^30^. For this reason, we did not apply these ITA skin tone bins in our analysis and instead used ITA as a continuous variable. Furthermore, the practical role of colorimetric devices in routine practice remains uncertain, though these devices offer several advantages that support their potential integration into clinical and research workflows. These include standardized outputs, high precision, and portability^28^. In the present study, device calibration and single-site measurement required only a few seconds of acquisition time and minimal staff training, demonstrating ease of use and time efficiency. However, colorimeters are still limited by cost, restriction to in-person use, and a lack of robust validation in diverse clinical contexts, which may restrict their broader adoption^10^. Nonetheless, the precision and objectivity of colorimeter devices, in addition to their decreasing cost over time, merit further investigation to define its optimal use in supporting reproducible, granular skin tone annotation in clinical and research settings.

A major bottleneck to building well-labeled datasets is that skin tone is seldom documented in routine clinical practice, leaving training data for AI models to be sourced retrospectively from clinical archives^31,32^. Photography-based labeling varied by lighting settings across all label types, limiting its generalizability. For TBP-based imaging, WL imaging tended to bias skin tone perception toward lighter shades, whereas XP imaging showed only a slight bias toward darker tones, more closely approximating in-person assessments. While TBP offers standardized imaging and lighting conditions of the entire body surface compared with 2D clinical photographs, it can still alter perceived skin tone, introduce anatomic site–specific shadowing, and remains largely restricted to academic centers, further decreasing the generalizability of subjective retrospective labeling in photography-based applications^33^. Manual labeling of images is likely affected by lighting conditions, photo quality, and annotator characteristics, so prospective labeling at the time of image acquisition is still critical^11,34–36^.

Automated approaches to estimating skin tone, including pixel-wise extraction of color from dermoscopic images, have been explored^37^. However, we observed systematic disagreement between dermoscopic image-extracted ITA values and in-person colorimeter-based ITA, likely due to variations in dermoscopy mode (polarized vs. non-polarized) and device-specific color processing. Additional obstacles, including image quality and artifacts such as dark corners, stickers, and pen markings, further undermine reliability^37^. On the other hand, crowdsourcing human annotations offers a rapid, cost-effective option for generating labels at scale^36,38–40^. Yet, in our FST labeling of dermoscopic images, we found limited accuracy with variability across body sites and dermoscopy modalities. Given the multitude of factors that can distort perceived skin tone in images, caution is warranted when labeling skin tone based on dermoscopic images alone.

Furthermore, labeling tools must incorporate distinct yet representative skin tone categories to effectively capture variations and detect biases in AI performance. Our clustering analysis revealed that MST groupings were slightly more coherent on the colorimetric space than Pantone. FST displayed the greatest dispersion, likely because photosensitivity does not reliably map onto actual skin color. It is important to note, however, that all subjective skin tone assessment tools demonstrated weak clustering overall, including MST. Moreover, MST binning showed a more consistent divergence in benign algorithm score functions compared with FST, suggesting it may be a more sensitive proxy for benchmarking algorithm fairness. Although prior work highlights poorer AI performance on darker skin types (FST V-VI), few have directly compared how various annotation methods affect these disparities^3^. Benčević et al. showed that segmentation performance can be biased under manual FST, image-based ITA estimation, and binary light or dark designations, yet they did not make direct comparisons by skin labeling method^41^. If skin tone indeed creates a clinically meaningful difference in image classifier performance, our findings suggest that MST may proactively identify subgroups at risk for misclassification and misdiagnosis, although further validation remains necessary.

Lastly, it is important to acknowledge that subjective skin tone assessments are shaped by sociocultural factors, including implicit bias, racial stereotypes, and colorism^42^. Culturally ingrained preferences for lighter or darker skin tones, which can vary by geographic region and ethnic context, can cause raters to over- or underestimate actual skin tone. For instance, race and skin tone are often inaccurately conflated, with studies showing that perceived race can systematically distort assessments of skin lightness^43,44^. Schumann et al. also demonstrated that MST annotations varied significantly by annotator geographic region, highlighting the influence of regional cultural context on perception of skin tone^27^. Similarly, Cook et al. found that the race of both the rater and subject affected MST and FST classifications, even when objective skin lightness was kept constant. In their study, subjective skin tone misclassification was influenced by factors such as racial cues, lip color, background contrast, and device lighting conditions. In contrast, colorimetry-based assessments were notably less affected by confounding variables^45^. Taken together, limitations of subjective tools emphasize the need for more objective methods, such as colorimetry, to reduce cultural and perceptual biases in skin tone classification.

Our study has several limitations. Not all sites were measured with colorimetry as the device was unavailable at the start of the study, reducing the sample size of these measurements. Despite efforts to standardize room conditions for in-person labeling, shadows may have introduced variability, particularly for lower extremity sites. Color values specific to devices, such as dermoscopic image-extracted ITA or SkinColorCatch colorimeter (Delfin Technologies, Kuopio, Finland) readings, may also limit the generalizability of our results. Further, images were stored in JPEG format which led to image compression and may have affected color fidelity. Moreover, this was a single-center study; multicenter efforts with larger cohorts are needed to validate our findings. Soft clustering approaches, as opposed to the hard clustering methodology used in our study, may also be explored in future research to better capture continuous skin tone variation. Nevertheless, this study is among the first to prospectively explore in-person and photography-based skin tone assessments, providing direct comparisons across multiple labeling tools.

Overall, there is a need for continued refinement and validation of skin tone labeling for medical AI applications, ensuring that non-invasive technologies are effective for individuals of all skin tones^3^. Our work adds to this emerging field by providing an in-depth exploration of in-person and photography-based labeling methods, addressing notable gaps in understanding how best to implement skin tone metrics for real-world AI. An important and novel product of this study is a skin-tone–annotated dataset that can support future AI development and improve model fairness^40^. Publicly available datasets with skin tone annotations remain sparse^3,4,36,46^, and more image datasets with skin tone labeling are needed to further this important area of research. Still, we highlight the limitations of currently available skin tone measurement tools, reinforcing the need for new, reliable annotation methods that are transparently developed based on diverse populations and validated across multiple real-world settings.

## Methods

### Patient recruitment

This prospective, single-center observational study was conducted at Memorial Sloan Kettering Cancer Center (New York, NY) from March 22, 2023, through November 22, 2024. The study was approved by the Institutional Review Board (IRB protocol #21-019). Written informed consent was obtained prior to study participation. Adult patients (over 18 years) scheduled to undergo a full-body skin examination by a dermatologist were eligible for recruitment via phone call within 2 weeks of their visit or approached in the clinic immediately after their dermatology visit. Consented patients were eligible for study inclusion if they presented with up to 13 skin lesions. In the clinic, a brief spoken survey was administered by a research staff member to obtain the participant’s self-reported race and ethnicity.

### Site selection

In each patient, up to 13 lesions were selected by the researcher from anywhere on the body and marked with an arrow sticker. Lesion size ranged from 2 to 12 mm in diameter to ensure that the lesion fits within the aperture of the dermatoscope. The number of lesions chosen depended on the patients’ time constraints. Best effort was made to include lesions from varied anatomic locations. The size and specific anatomic location of each lesion were recorded. Inflammatory conditions, such as rashes, were excluded. Non-lesional sites were also marked at 11 standardized locations, including the mid forehead, upper mid chest, mid abdomen, right inner upper arm, right inner wrist, right dorsal forearm, mid upper back, mid lower back, right shin, right calf, and right lateral sole (Fig. 3a).

### In-person skin tone assessment

All in-person skin tone evaluations were done in a consistent room with standardized lighting, temperature, and humidity. Patients rested for at least 5 min prior to skin tone assessments. Fitzpatrick Skin Type (FST) was determined by asking the question, “If you were to be outdoors without sunscreen for 1 h, please describe how well or poorly you burn and tan.” The patients’ dermatologist interpreted these answer responses and categorized patients as one of the following FST categories: I (always burns, does not tan), II (burns easily, tans poorly), III (tans after initial burn), IV (burns minimally, tans easily), V (rarely burns, tans darkly easily), VI (never burns, always tans darkly)^7^. FST was uniformly assigned to all skin sites (lesional and non-lesional) associated with the patient.

Two handheld, visual skin tone scales were used to assess skin tone. The Pantone SkinTone Guide (Pantone; [Pantone LLC, Carlstadt, NJ]) consists of 138 unique skin tone swatches that vary by hue (1–5), undertone (‘Y’ [yellow] or ‘R’ [red]), and lightness (1 [lightest] to 15 [darkest])^10,12^. The Monk Skin Tone (MST) scale is an open-source tool consisting of 10 shaded orbs created for image-based and in-person skin tone labeling^9–11^. Two independent raters assessed in-person skin tone using Pantone and MST at all marked lesional and non-lesional sites.

Triplicate measurements were taken using the SkinColorCatch colorimeter (Delfin Technologies, Kuopio, Finland) on all 11 non-lesional body sites in 47 patients. The colorimeter was applied gently to the skin to avoid pressure or blanching. The skin color metrics collected from the colorimeter included L*a*b* color values (L*: luminance [range 0 (black) to 100 (white)], a*: red/green value [range −127 (green) to +127 (red)], b*: blue/yellow value [range −127 (blue) to +127 (yellow)]). Individual typology angle (ITA) metrics were automatically calculated from L*a*b*. However, colorimetry could not be performed at all sites because the device was unavailable at the start of the study due to pending IRB approval.

### Image acquisition

For all patients, three-dimensional (3D) total body photography (TBP) was taken under both cross-polarized (XP) and white light (WL) settings using the VECTRA WB360 photography system (Canfield Scientific, Parsippany, NJ, USA). Patients were then randomized to imaging of their lesional and non-lesional sites with either the Canon Electro-Optical System (EOS) Rebel T6i single-lens reflex (SLR) dermatoscope (Canon Inc, Tokyo, Japan) or the 7th generation iPod Touch (Apple Inc, Cupertino, CA, USA; software version 15.8.3) with the VEOS DGX mobile application (version 2.0.2) and Canfield VEOS dermoscopy attachment (Canfield Scientific). The iPod Touch was used for 31 patients, and imaging parameters were automatically controlled by the VEOS application with white balance calibrated using a white card at study initiation. The Canon EOS Rebel was used for 33 patients, and images were acquired using fixed exposure with white balance calibration at study initiation. Images were taken for each participant in 4 settings: polarized contact (P-C), polarized non-contact (P-NC), non-polarized contact (NP-C), non-polarized non-contact (NP-NC). To capture contact dermoscopy photos, alcohol was sprayed on the skin site and a dermoscopic glass lens was gently pressed against the skin. DermaGraphix imaging software (Canfield Scientific) was used to tag all lesional and non-lesional sites to enable attribution of dermoscopic images to their respective locations.

### 3D TBP-based skin tone assessment

Following a washout period of 4 weeks after in-clinic skin tone assessment, Rater 1 assessed skin tone on 3D TBP digital skin map using the VECTRA image database for all non-lesional body sites using a digital MST scale. Photos were zoomed to approximately real-life size and the MST scale orb diameter used was scaled to ~1 inch. Participants were randomized to retrospective assessment of their 3D TBP using either XP or WL settings. After another washout period of 2 weeks, the TBP digital skin map taken with the other lighting setting was assessed using digital MST.

### Crowdsourced photography-based FST assessment

Prior to initiating crowdsourced annotations of FST, all prospectively acquired dermoscopy images of skin lesions underwent manual quality assurance review. Images were excluded if they were duplicates, contained identifiable patient information, or exhibited poor quality due to significant blur, faulty lighting, or obscuring artifacts.

To obtain non-expert annotations of FST, we utilized DiagnosUS (Centaur Labs, Boston, MA, USA), a commercially available mobile application, through a collaboration agreement. This app allows registered users to participate in annotation competitions with monetary prizes awarded to those demonstrating the highest annotation accuracy, which enhances engagement and annotation quality.

Before starting the annotation task, 1327 users completed a survey asking about their level of medical training, specialty of interest or practice, race and ethnicity, and the country of residence. Following the survey, users were required to complete a tutorial that provided a brief overview of the FST scale and the purpose of the annotation task. The tutorial included sample dermoscopy images of skin lesions representing all FSTs under all dermoscopy lighting modes. Users also participated in practice exercises that provided immediate feedback on their responses. During the tutorial, users were informed that FST labels may vary depending on dermoscopy lighting mode, anatomical site, and background photodamage.

To ensure annotation quality, users were required to maintain a trailing average accuracy ranking within the top 80th percentile of participants for their annotations to be considered “qualified reads.” Each image was annotated by multiple users, and the “majority label” was defined as the FST label selected by at least 70% of at least 3 qualified reads for that image or the label selected by the majority if more than 12 qualified reads were collected. In total, 1327 users provided FST annotations of our dermoscopic images via the DiagnosUS mobile application. Of these users, 422 (31.8%) were medical students, 221 (16.7%) undergraduate students, 49 (3.7%) resident physicians, 62 (4.7%) nurse practitioners, and 20 (1.9%) attending physicians (Supplementary Table S3). Users annotated 2404 images of 618 lesions, generating a total of 65,024 qualified reads. The crowd-sourced majority labels were subsequently compared with in-person FST labels to assess overall accuracy.

### Extraction of ITA from dermoscopy images

All dermoscopy photos were stored in JPEG format, encoding color data in the standardized RGB color space. L*a*b* values were extracted from image files using *Pillow*, *NumPy*, *scikit-image*, and standard Python libraries. Median pixel-wise RGB values were extracted from each dermoscopic image of all non-lesional sites. These RGB values were then linearized, transformed to XYZ color space, and then converted to L*a*b*. ITA values were derived from L* and b* values using the formula: ITA = arctan((L* − 50)/b*) × (180/π).

### ADAE AI algorithm description

All patients underwent comprehensive skin examinations by a dermatologist immediately prior to recruitment, so lesions selected for assessment were assumed to be benign, as suspicious lesions had already been biopsied. Inclusion criteria for this analysis were: images of the P-C mode, images of lesion sites, 1 image per site, and images that can be made publicly available through the International Skin Imaging Collaboration (ISIC) Archive. A total of 587 lesional images were analyzed. By FST, the images were distributed by the following: I (*n* = 92), II (*n* = 110), III (*n* = 101), IV (*n* = 91), V (*n* = 94), VI (*n* = 99). By MST, the images were distributed by the following: 1 (*n* = 53), 2 (*n* = 123), 3 (*n* = 131), 4 (*n* = 64), 5 (*n* = 64), 6 (*n* = 52), 7 (*n* = 62), 8 (*n* = 27), 9 (*n* = 11). Scoring of benign lesions was assessed using the melanoma classifier ADAE algorithm, the top performing algorithm in the Society for Imaging Informatics in Medicine-International Skin Imaging Collaboration (SIIM-ISIC) 2020 Melanoma Classification Challenge hosted by Kaggle, which used a convenience test set of 10,982 public dermoscopy images from 6 international dermatology centers^16,19,42^. ADAE achieved an overall area under the curve (AUC) of 0.9490. This algorithm uses a set of 18 prediction models (16 using EfficientNet and 2 using ResNet architecture), with 4 models that incorporate clinical metadata. Each prediction model was trained using 5-fold cross-validation, resulting in a total of 90 fold-level scores that were log transformed and averaged^19^. The ADAE score provides a malignancy risk score (0–100%) where scores closer to 0 are more likely benign and those closer to 100% are likely malignant. The ADAE AI model is publicly available at the following GitHub repository: https://github.com/ISIC-Research/ADAE.

### Statistics

Cohorts for all sub-analyses are defined in Table 1. All deidentified data used in this study are publicly available at 10.34970/962049. Data analysis was performed in R version 4.4.2 (2024-10-31) across different workspaces.

#### For in-person skin tone assessments

Inter-rater reliability was assessed using linear-weighted Cohen’s kappa (κ) for the Pantone and MST scales in a cohort of patients who had 2 ratings for both scales (*n* = 607 lesional sites, *n* = 639 non-lesional sites), as appropriate for ordered categorical data. Agreement for Pantone was stratified by pigment (1–15) and undertone (5 R [red]-5Y [yellow]). ICC was used to assess agreement between colorimeter triplicate measurements of ITA (*n* = 502 non-lesional sites), as appropriate for continuous variables. Agreement analyses were performed in R using *irr* for weighted kappa statistics and *psych* for ICC. FST, MST, and Pantone categories assessed in-person were compared according to their degree of clustering within the 2-dimensional (2D) CIELAB color space, utilizing objective colorimeter measurements. In this analysis, average L* and average b* measurements obtained by the colorimeter were considered ground truth (2D estimate) for skin color. Only non-lesion sites (*n* = 501 sites across 47 patients) that were assessed with colorimetry and had at least 1 MST rating and at least 1 Pantone rating were included and considered independent observations. One rating was selected at random in sites where both raters provided a rating for a given scale. The degree by which in-person human skin tone classification categories group together was quantified using 2 measures of clustering (Fig. 1A–C): the Rousseeuw Silhouette Index (RSI), which focuses on the balance between intra-cluster cohesion and inter-cluster separation, and the Davies-Bouldin Index (DBI), which evaluates clustering compactness and separation^47,48^. In such instances of single-observation clusters, the Silhouette value of those observations was assumed to be 1 in the computation of RSI. DBI was calculated using Euclidean distances. The CIE (L*,b*) analysis of skin-tone class defined-clusters was executed in R with the *clusterSim* and *tidyverse* packages.

#### For photography-based skin tone assessments

Using the MST scale, Rater 1 assessed non-lesional sites first in-clinic, then retrospectively via 3D TBP under XP (*n* = 563 non-lesional sites) and WL (*n* = 566 non-lesion sites) settings. Linear weighted κ was used to assess agreement between Rater 1’s in-person MST and TBP-based MST assessments. ICC was calculated to compare colorimeter-derived ITA to image-extracted ITA values (*n* = 474 non-lesional sites). Differences between in-person and TBP-based MST ratings were also assessed using paired t-tests.

To compare agreement between in-person FST assessments and crowdsourced FST labels of lesional sites, linear weighted κ test was also performed with FST as ordered categorical variable. Only lesions with available images were included (*n* = 618 lesional sites). A Chi-squared test was performed to assess whether crowdsourced FST labels were significantly influenced by dermoscopy mode or anatomical site of the lesion. A *P*-value of <0.05 was considered statistically significant.

#### For ADAE classifier performance

ADAE AI classifier performance was evaluated using the 2-sample Kolmogorov-Smirnow (KS) test to assess differences in score distributions between FST and MST annotations as well as mean algorithm scores by skin tone subgroup. Lesion distributions are outlined in Supplementary Table S4. KS statistics were computed using the R *stats* package for comparing ADAE score distributions.

## Supplementary information

Supplementary Information

## Data Availability

All experimental data (including dermoscopy images) needed to reproduce this study have been deidentified and are available at 10.34970/962049. 3D TBP is unavailable due to patient privacy given the sensitive nature of the images. The ADAE AI model is publicly available in the following GitHub repository: https://github.com/ISIC-Research/ADAE.

## Code availability

Open-source software as detailed in the Methods: *L*a*b** summaries were extracted from image files using *Pillow*, *NumPy*, *scikit-image*, and standard Python libraries. The *L*a*b** cluster analysis was executed in R with the *clusterSim* and *tidyverse* packages. KS statistics were computed using the R *stats* package for comparing ADAE score distributions.
