# Supplementary material for: Evaluating skin tone scales for dermatologic dataset labeling: a prospective-comparative study
Source: NPJ Digit Med. 2025 Dec 22;8:787. doi: 10.1038/s41746-025-02245-2 (PMC12749783; doi:10.1038/s41746-025-02245-2)
Supplement: Supplementary file 1 — Supplementary Information [file 41746_2025_2245_MOESM1_ESM.pdf]

Supplementary Information

Table S1: Cluster analysis demonstrating the Rousseeuw's Silhouette index (RSI), Davies-Bouldin index (DBI), and per-cluster dispersion measures for Fitzpatrick Skin Type (FST), Monk Skin Tone (MST), and Pantone measurements

Classifier	RSI[a]	DBI[b]	Per-cluster Dispersion Measure
**FST**	-0.0467	10.59	I: 5.92<br>II: 6.65<br>III: 7.23<br>IV: 8.19<br>V: 8.99<br>VI: 9.10
**MST**	-0.0406	2.70	1: 6.41<br>2: 5.97<br>3: 6.21<br>4: 6.57<br>5: 5.48<br>6: 4.88<br>7: 5.56<br>8: 5.14<br>9: 3.77<br>10: NA
**Pantone**	-0.5088	7.59	-

[a]*RSI (range: [-1, 1]) measures the balance between intra-cluster cohesion and inter-cluster separation, with higher values indicating greater clustering. MST demonstrated the best clustering with the highest RSI value of -0.0406, suggesting more coherent groupings compared with FST and Pantone. However, the RSI was close to 0 for all 3 skin tone measurement scales, implying weak clustering even for MST.*
[b]*DBI (range: [0, inf)) assesses clustering compactness and separation, with lower values indicating greater clustering. MST had the lowest DBI value (2.70), reflecting stronger clustering compared with FST (10.59) and Pantone (7.59). The per-cluster dispersion measure evaluates the spread within each cluster. MST displayed a relatively consistent dispersion across its categories, with the tightest grouping for MST 9 (3.77). In contrast, FST showed greater dispersion, particularly in higher FST categories, suggesting less uniformity in skin tone classification.*

Figure S1: Anatomical site-specific cluster dispersion measures quantifying the association between colorimeter derived CIE (*L, *B**) and each respectively administered skin tone scale (MST, FST, and Pantone).**

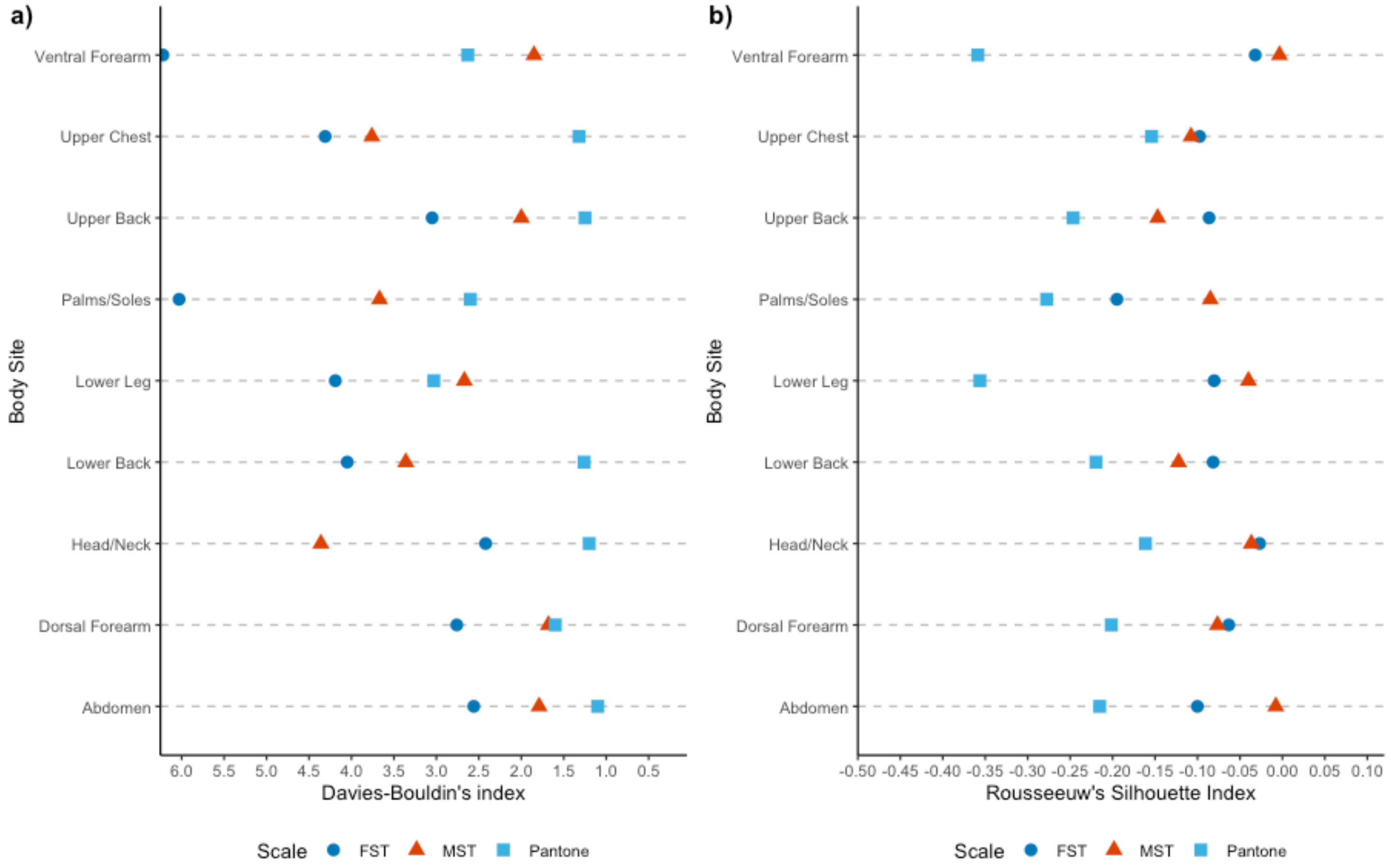

a, Davies-Bouldin's index (range: [0, inf)) assesses clustering compactness and separation, with lower values indicating greater clustering. MST and Pantone tended to score better than FST in most analyzed body sites. **b,** Rousseeuw's Silhouette index (range: [-1, 1]) measures the balance between intra-cluster cohesion and inter-cluster separation, with higher values indicating greater clustering. Pantone scored the lowest on all analyzed body sites, while FST and MST tended to score similarly.

Figure S2: Confusion matrices displaying percent concordance of photography-based versus in-person skin tone assessments.

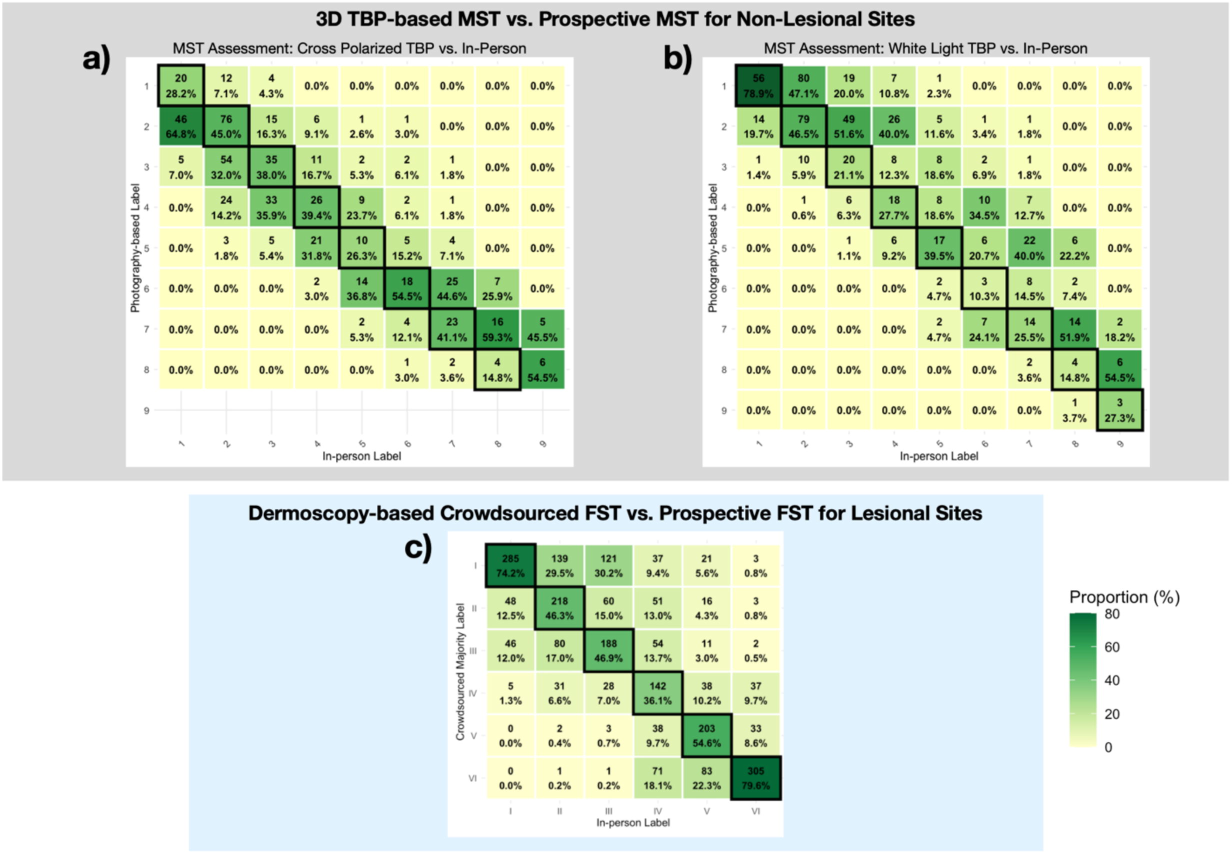

Diagonal cells (outlined in black) represent concordant classifications and off-diagonal cells show the frequency of misclassifications. **a,** Confusion matrix comparing total body photography (TBP)-based Monk Skin Tone (MST) labels using cross polarized (XP) lighting compared with in-person MST. Number of non-lesional sites per in-person MST category: 1 (*n* = 71), 2 (*n* = 169), 3 (*n* = 92), 4 (*n* = 66), 5 (*n* = 38), 6 (*n* = 33), 7 (*n* = 56), 8 (*n* = 27), 9 (*n* = 11). **b,** Confusion matrix comparing TBP-based MST labels using white light (WL) to in-person MST. Number of non-lesional sites per in-person MST category: 1 (*n* = 71), 2 (*n* = 170), 3 (*n* = 95), 4 (*n* = 65), 5 (*n* = 43), 6 (*n* = 29), 7 (*n* = 55), 8 (*n* = 27), 9 (*n* = 11). **c,** Confusion matrix comparing crowdsourced dermoscopy-based FST labels to in-person FST labels. Number of lesional images per in-person FST: (*n* = 384), II (*n* = 471), III (*n* = 401), IV (*n* = 393), V (*n* = 372), VI (*n* = 383).

Figure S3: Differences in luminance (*L) and yellow chromaticity (*b**) values measured in-person using the colorimeter vs. extracted from dermoscopic images (labeled by dermoscopic mode).**

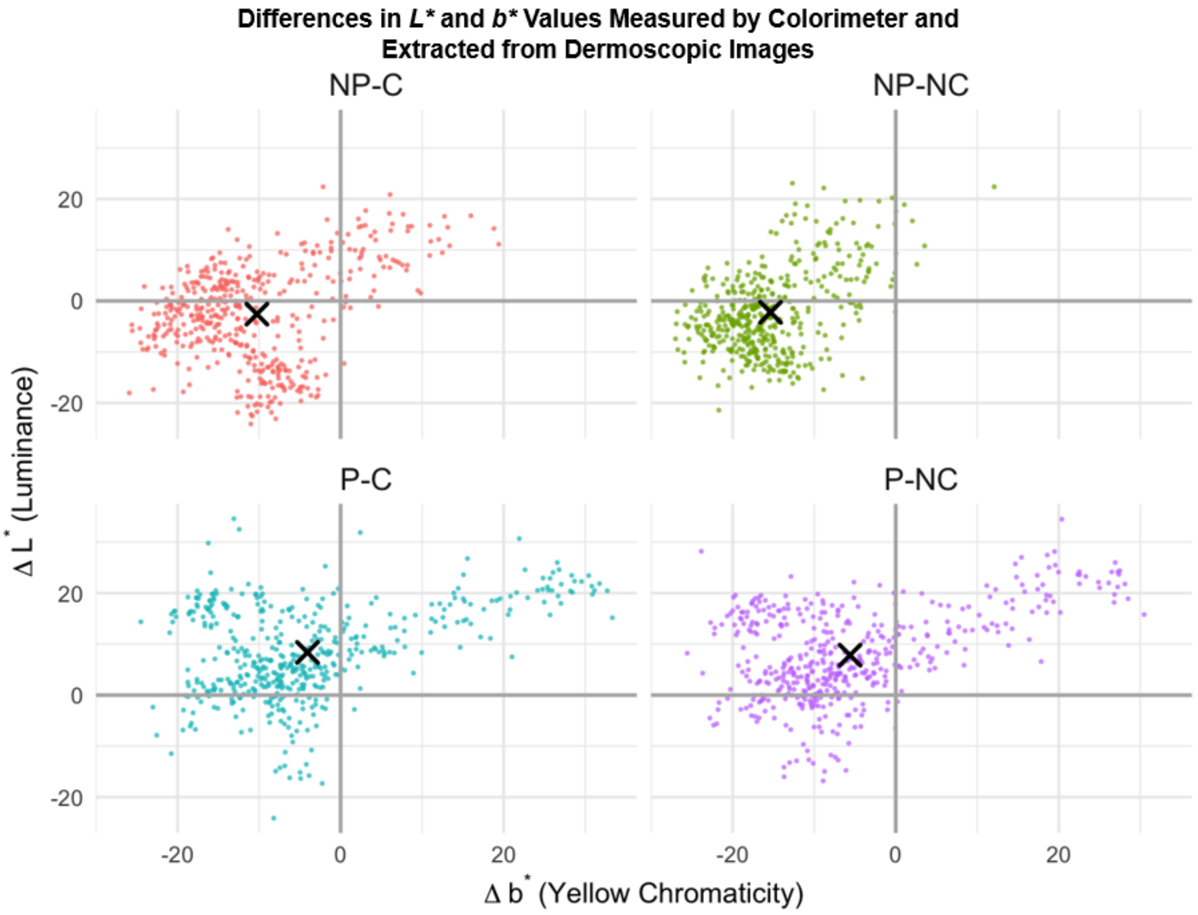

The difference between colorimeter versus image-extracted values for luminance and yellow chromaticity was calculated as follows: ΔL^* = (image-extracted L^*) - (colorimeter-derived L^*). Δb^* = (image-extracted b^*) - (colorimeter-derived b^*). Each colored point represents an individual measurement signified by the (ΔL^*, Δb^*) coordinate. Positive ΔL^* and Δb^* values represent lighter shades and more yellow hues, respectively. Negative ΔL^* and Δb^* values represent darker shades and less yellow (bluer) hues, respectively. The centroids (black "X" markers) indicate the average discrepancy in each dermoscopic mode. In general, non-polarized contact (NP-C) and non-contact (NP-NC) modes tend to show negative differences in L^* and b^*, indicating darker and less yellow hues compared with colorimeter measurements. Polarized contact (P-C) and non-contact (P-NC) tend to exhibit positive differences in L^* and negative differences in b^*, indicating lighter and less yellow hues than colorimeter measurements.

Figure S4: Skin tone qualitatively differs within the same single individual by body site or within the same lesion depending on dermoscopic mode.

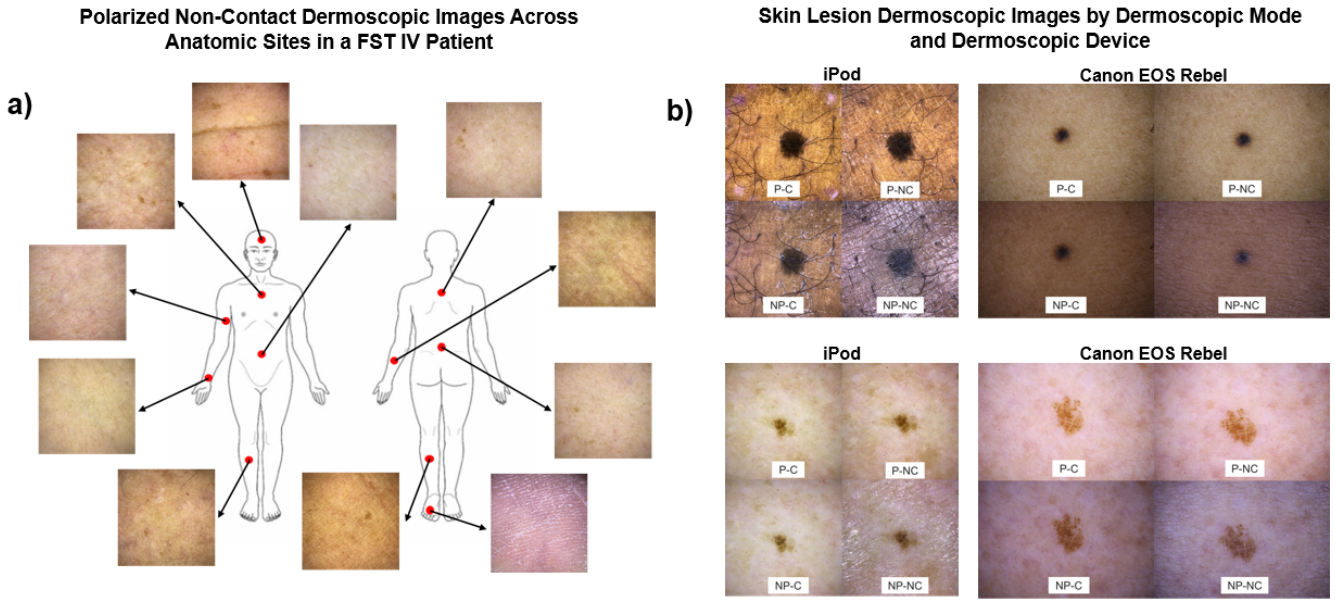

a, Visual example of polarized non-contact dermoscopy photos taken from a patient classified as having Fitzpatrick Skin Type (FST) IV demonstrating differences in skin color across the eleven standardized non-lesional sites, signified by red dots. **b,** Examples of skin lesions imaged with 4 different dermoscopy modes: P-C (polarized contact), P-NC (polarized non-contact), NP-C (non-polarized contact), NP-NC (non-polarized non-contact). Top left: FST VI taken with 7th generation iPod Touch (Apple Inc, Cupertino, CA, USA; software version 15.8.3). Bottom left: FST II taken with iPod Touch. Top right: FST V taken with Canon EOS (Electro-Optical System) Rebel T6i single-lens reflex (SLR) dermatoscope (Canon Inc, Tokyo, Japan). Bottom right: FST II taken with Canon EOS Rebel.

Table S2: Matrices comparing Kolmogorov-Smirnov (KS) statistic[*] across all observed values of All Data Are Ext algorithm (ADAE)-score by in-person skin tone assessment

a: Matrix comparing KS statistic across all observed values of ADAE score by Fitzpatrick Skin Type (FST)

**FST**	**I**	**II**	**III**	**IV**	**V**	**VI**
**I**	0	0.159	0.169	0.194	**0.210**	0.179
**II**		0	0.081	0.080	0.125	**0.245**
**III**			0	0.087	0.087	**0.289**
**IV**				0	0.121	**0.311**
**V**					0	**0.321**
**VI**						0

b: Matrix comparing KS statistic across all observed values of ADAE score by Monk Skin Tone (MST)

**MST**	**1**	**2**	**3**	**4**	**5**	**6**	**7**	**8**	**9**
**1**	0	0.063	0.086	0.155	0.112	0.346	0.181	**0.396**	**0.642**
**2**		0	0.078	0.154	0.101	0.185	0.196	**0.415**	**0.659**
**3**			0	0.150	0.119	0.157	0.188	**0.397**	**0.695**
**4**				0	0.094	0.238	0.217	**0.438**	**0.797**
**5**					0	0.204	0.181	**0.469**	**0.734**
**6**						0	0.241	0.288	**0.596**
**7**							0	**0.465**	**0.726**
**8**								0	**0.407**
**9**									0

[*] *The KS statistic is used to measure differences in ADAE malignancy scores across skin tone categories. The closer the KS statistic is to 1, the larger the difference in the ADAE score distributions. Tests associated with P-value < 0.05 are emphasized with bolded and underlined text. MST generally reveals larger differences in ADAE score distributions compared with FST, and statistically significant differences highlight MST's greater sensitivity in detecting disparities in artificial intelligence (AI) performance across skin tone.*

Table S3: Demographics of participants in the crowdsourced Fitzpatrick Skin Type (FST) annotation task

	**Annotators (*n* = 1,327)**
**Level of training, *n* (%)**	
Medical student	422 (31.8%)
Undergraduate student	221 (16.7%)
Other healthcare student	101 (7.6%)
Nurse practitioner	62 (4.7%)
Resident physician	49 (3.7%)
Physician assistant	20 (1.9%)
Attending physician	20 (1.9%)
Other	432 (32.6%)
**Specialty of Interest or Practice, *n* (%)**	
Surgery	125 (9.4%)
Dermatology	98 (7.4%)
Family Medicine	91 (6.9%)
Internal Medicine	73 (5.5%)
Other or not applicable	1,013 (76.3%)
**Race/Ethnicity, *n* (%)**	
Asian	511 (38.5%)
Black or African American	422 (31.8%)
Hispanic or Latino or Spanish origin of any race	69 (5.2%)
Non-Hispanic White	67 (5.0%)
American Indian or Alaskan Native	14 (1.1%)
Native Hawaiian or Other Pacific Islander	10 (0.8%)
Mixed	34 (2.6%)
No response	200 (15.1%)
**Country of Origin, *n* (%)**	
Philippines	295 (22.2%)
Ghana	200 (15.1%)
Nigeria	167 (12.6%)
Pakistan	100 (7.5%)
South Africa	77 (5.8%)
Egypt	42 (3.2%)
India	43 (3.2%)
United States of America	29 (2.2%)
Other	69 (32.9%)

Table S4: Distribution of lesional sites with available dermoscopy images by in-person Fitzpatrick Skin Type (FST) across all in-person Monk Skin Tone (MST) shades* and anatomical body sites

	I (n = 101)	II (n = 118)	III (n = 101)	IV (n = 100)	V (n = 100)	VI (n = 101)	Overall (n = 621)
**MST**							
1	24 (23.8%)	4 (3.4%)	9 (8.9%)	0 (0%)	0 (0%)	0 (0%)	37 (6.0%)
2	60 (59.4%)	50 (42.4%)	42 (41.6%)	9 (9.0%)	3 (3.0%)	0 (0%)	164 (26.4%)
3	10 (9.9%)	43 (36.4%)	38 (37.6%)	21 (21.0%)	5 (5.0%)	0 (0%)	117 (18.8%)
4	0 (0%)	4 (3.4%)	12 (11.9%)	27 (27.0%)	10 (10.0%)	10 (9.9%)	63 (10.1%)
5	0 (0%)	7 (5.9%)	0 (0%)	16 (16.0%)	20 (20.0%)	9 (8.9%)	52 (8.4%)
6	0 (0%)	0 (0%)	0 (0%)	16 (16.0%)	32 (32.0%)	21 (20.8%)	69 (11.1%)
7	0 (0%)	0 (0%)	0 (0%)	5 (5.0%)	24 (24.0%)	30 (29.7%)	59 (9.5%)
8	0 (0%)	0 (0%)	0 (0%)	0 (0%)	5 (5.0%)	25 (24.8%)	30 (4.8%)
9	0 (0%)	0 (0%)	0 (0%)	0 (0%)	0 (0%)	5 (5.0%)	5 (0.8%)
10	0 (0%)	0 (0%)	0 (0%)	0 (0%)	0 (0%)	0 (0%)	0 (0%)
NA	7 (6.9%)	10 (8.5%)	0 (0%)	6 (6.0%)	1 (1.0%)	1 (1.0%)	25 (4.0%)
**Body Site**							
Anterior torso	22 (21.8%)	24 (20.3%)	17 (16.8%)	18 (18.0%)	16 (16.0%)	16 (15.8%)	113 (18.2%)
Head/neck	4 (4.0%)	5 (4.2%)	4 (4.0%)	9 (9.0%)	12 (12.0%)	9 (8.9%)	43 (6.9%)
Lateral torso	5 (5.0%)	0 (0%)	3 (3.0%)	1 (1.0%)	1 (1.0%)	0 (0%)	10 (1.6%)
Lower extremity	11 (10.9%)	13 (11.0%)	13 (12.9%)	24 (24.0%)	15 (15.0%)	24 (23.8%)	100 (16.1%)
Posterior torso	31 (30.7%)	41 (34.7%)	35 (34.7%)	27 (27.0%)	35 (35.0%)	27 (26.7%)	196 (31.6%)
Upper extremity	28 (27.7%)	35 (29.7%)	28 (27.7%)	20 (20.0%)	20 (20.0%)	18 (17.8%)	149 (24.0%)
Palms/soles	0 (0%)	0 (0%)	1 (1.0%)	1 (1.0%)	1 (1.0%)	7 (6.9%)	10 (1.6%)

**MST shades for each lesional site were averaged between the 2 raters. If the average was a non-integer, it was rounded in the direction of the patients' MST shade averaged across all lesional sites.*
